# Artificial intelligence in nursing: advancing clinical judgment and decision-making

**DOI:** 10.1590/0034-7167.2025780401

**Published:** 2025-08-08

**Authors:** Luciano Magalhães Vitorino, Gerson Hiroshi Yoshinari, Luís Carlos Lopes-Júnior

**Affiliations:** I Faculdade de Medicina de Itajubá. Itajubá, Minas Gerais, Brazil; II Universidade Federal do Espírito Santo. Vitória, Espírito Santo, Brazil

The rapid evolution of artificial intelligence (AI) is reshaping healthcare by automating routine tasks, streamlining workflows, and enhancing clinical decision-making. Advanced computational models are now capable of analyzing large-scale vast clinical datasets and generate real-time evidence-based recommendations. Despite promising performance in controlled research settings, a substantial gap remains in the prospective validation of AI applications within real-world nursing environments. Current literature suggests that while AI holds significant potential to support nurses in addressing both hospital and primary care challenges, essential human competencies, such as clinical reasoning and reflective decision-making, remain irreplaceable ^(1-4)^.

Rony *et al*.^(5)^ demonstrate how AI algorithms can be used to monitor vital signs and detect early signs of patient deterioration, thereby enabling nurses to allocate more time to direct patient care. Their findings highlight that automated data analysis can support clinical efficiency and patient safety by reducing nurses’ cognitive load for routine monitoring. However, the authors emphasize that AI-generated recommendations must undergo rigorous human oversight and clinical judgment prior to integration into standard nursing practice.

A range of AI applications in nursing has already been identified, with machine learning models currently being employed for fall prediction, pressure injury risk assessment, and care coordination. These technologies hold significant potential to optimize clinical workflows and improve patient outcomes across both acute care and community settings. Nevertheless, the limited number of prospective studies and the scarcity of real-world validation underscore the urgent need for further research to ensure the clinical reliability and safety of these emerging tools^(2,5,6)^.

In the field of natural language processing (NLP), Hossain *et al*.^(3)^ examine the extraction of clinical insights from electronic health records (EHRs). Their findings demonstrates that NLP can efficiently transform unstructured clinical texts, such as nursing notes and patient histories, into actionable data. By automating the interpretation of free-text documentation, AI systems help reduce administrative burdens and allow nurses to devote more attention to clinical decision-making. Yet the study also underscores ongoing challenges, including data imbalance and the need for rigorous data curation, which must be addressed to ensure the reliability and accuracy of NLP outputs^(2,3,7)^.

It is imperative that we explore the opportunities and challenges associated with the integration of AI into nursing education and practice, emphasizing the potential of personalized learning systems to tailor content to individual learners and thereby enhance both academic achievement and clinical performance. While AI can assist by automating tasks such as documentation and resource allocation, we maintain that the ultimate responsibility for patient care must remain with trained healthcare professionals. This perspective reinforces the need for continuous professional development and establishment of robust ethical guidelines to ensure patient safety and uphold clinical decision-making integrity^(1)^.

Porcellato *et al*.^(6)^ present a systematic review of AI applications in critical care nursing, highlighting the use of deep learning and machine learning techniques to predict complications, support triage, and monitor critical vital signs in Intensive Care Units and emergency departments. While these models demonstrate promising levels of accuracy, the heterogeneity in study designs and data sources limits the generalizability of the findings. The authors advocate for robust interventional studies to assess the true impact of AI on nursing-sensitive outcomes^(3,6)^.

Topol^(2)^ offers a visionary perspective on high-performance medicine by emphasizing the convergence of human and AI. He describes how modern AI systems are capable of processing high-resolution medical images, continuous biosensor data, and genomic information with exceptional speed and precision. However, he underscores that the interpretation of these outputs continues to rely on human expertise^(2)^. While AI has the potential to enhance diagnostic accuracy and improve operational efficiency, Topol highlights persistent concerns regarding algorithmic bias, data privacy, and the opaque, “black box” nature of deep learning systems. He advocates for a hybrid model in which human judgment and AI-driven insights are integrated, thus harnessing the complementary strengths of both^(2,3,5)^.

Passerini *et al*. highlight the importance of fostering effective collaboration between humans and large language models (LLMs)^(8)^. While LLMs demonstrate impressive performance across a wide range of language-based tasks, the authors note key limitations, including hallucinations, inconsistencies, and sycophancy responses. In parallel, emerging large multimodal models (LMMs) are beginning to extend these capabilities by integrating visual data analysis into clinical decision-making, such as interpreting an image of a scar to inform a treatment plan. Their work advocates for the development of structured frameworks for human-AI interaction, in which clinicians play a central role in guiding and validating AI-generated outputs. This approach aims to enhance clinical decision-making while avoiding overreliance on automation^(2,7,8)^.

We emphasized that AI tools have the potential to significantly enhance clinical decision-making by processing complex datasets and generating insights that support reflective clinical judgment. Nonetheless, while AI can strengthen data-driven decision-making, its use must remain embedded within a framework that upholds clinicians’ authority, autonomy, and critical thinking. Rigorous validation and continuous monitoring are essential to ensure accuracy, mitigate risks, and prevent potential errors^(9)^.

Moreover, within the context of Brazilian nursing, AI, especially NLP models such as ChatGPT, has the potential to streamline nursing care systematization and enhance administrative efficiency. However, we assert that clinical accountability must remain exclusively with licensed practitioners. We caution against the delegation of clinical responsibility to machines and advocate for the integration of AI education into nursing curricula to develop both technological proficiency and strong clinical judgment^(4)^.

Collectively, these studies illuminate the transformative potential of AI in nursing across variety of care settings. In hospital environments, AI can automate data analysis, optimize triage systems, and support rapid diagnostic decision-making, thereby enhancing operational efficiency and enabling nurses to focus on high-acuity clinical interventions. In primary care, AI-driven tools can analyze EHRs and forecast health trends, supporting preventive care and personalized patient management. Despite these promising applications, a critical gap persists: most current research remains retrospective or experimental, with limited validation in live-world clinical environments. Furthermore, the inherent risks associated with inaccurate or biased AI outputs reinforce the need for clinicians to maintain active oversight and apply critical judgment at all times.

The integration of AI and LLMs into nursing decision-making represents a promising and rapidly evolving frontier. Advanced models can provide preliminary risk assessments and concise data summaries, enabling clinicians to devote greater attention to complex, high-stakes decisions. Recent advancements in reasoning models, such as ChatGPT o3-series, DeepSeek R1, and Gemini flash thinking, offer improved contextual understanding and sophisticated reasoning capabilities, with the potential to transform the interpretation and application of clinical data. Nonetheless, these innovations still require rigorous prospective validation before they can be safely adopted in clinical practice. As these technologies continue to advance, human-AI collaboration must be intentionally designed to leverage the computational power of AI while preserving the essential human dimensions of empathy, ethical judgment, and personalized care. Accordingly, we underscore the importance of incorporating AI literacy into professional training programs and urge healthcare systems to develop clear, evidence-based guidelines to ensure the responsible and effective use of these tools.

We recognize that integrating AI into clinical practice presents significant ethical, legal, and curatorial challenges that must be carefully addressed. Mitigating algorithmic biases, safeguarding data privacy, and clarifying legal implications are critical to the safe and responsible implementation of AI in nursing. Ensuring the explainability and transparency of “black box” models is essential to maintaining trust in AI systems and their outputs. Equally important is the rigorous curation of training data to minimize biases and uphold patient confidentiality. Only through the establishment of stringent regulatory frameworks and continuous validation efforts can we ensure that AI systems produce reliable, unbiased, and interpretable results, supporting, rather than compromising, the quality and safety of patient care.

In conclusion, AI offers substantial opportunities to enhance efficiency, reduce administrative burdens, and improve patient outcomes across both hospital and primary care settings. However, its safe and effective implementation depends on robust prospective research, meticulous data curation, and continuous professional oversight. Bridging the gap between automated efficiency and clinical excellence requires addressing key ethical challenges, including bias mitigation, data privacy, and legal accountability, while increasing the transparency of “black box” models. Embracing a hybrid model, which assists but does not replace human clinical reasoning, is essential to ensuring that AI strengthens, rather than compromises, the quality and integrity of nursing care.
