# Supplementary material for: Artificial intelligence in nursing: advancing clinical judgment and decision-making
Source: Rev Bras Enferm. 2025 Aug 8;78(4):e780401. doi: 10.1590/0034-7167.2025780401 (PMC12334131; doi:10.1590/0034-7167.2025780401)
Supplement: Translation

# Translation: Inteligência Artificial na Enfermagem: avanços no julgamento clínico e na tomada de decisão

**DOI:** 10.1590/0034-7167.2025780401pt

**Authors:** Luciano Magalhães Vitorino, Gerson Hiroshi Yoshinari, Luís Carlos Lopes-Júnior

**Affiliations:** I Faculdade de Medicina de Itajubá. Itajubá, Minas Gerais, Brasil; II Universidade Federal do Espírito Santo. Vitória, Espírito Santo, Brasil

A rápida evolução da inteligência artificial (IA) tem remodelado o campo da saúde ao automatizar tarefas rotineiras, otimizar fluxos de trabalho e aprimorar a tomada de decisão clínica. Modelos computacionais avançados demonstram capacidade de analisar grandes conjuntos de dados clínicos e gerar, em tempo real, recomendações fundamentadas em evidências. No entanto, apesar do desempenho promissor em ambientes de pesquisa controlados, persiste uma lacuna significativa no que diz respeito à validação prospectiva das aplicações de IA em contextos reais da prática de enfermagem. A literatura atual sugere que, embora a IA apresente considerável potencial para apoiar enfermeiras(os) na resolução de desafios tanto hospitalares quanto na atenção primária à saúde, competências humanas essenciais-como o raciocínio clínico e a tomada de decisão reflexiva-permanecem insubstituíveis ^(1-4)^.

Rony, et al^(5)^ demonstram que algoritmos de IA podem ser utilizados para monitorar sinais vitais e detectar precocemente sinais de deterioração clínica, permitindo, assim, que profissionais de enfermagem dediquem mais tempo ao cuidado direto ao paciente. Seus achados evidenciam que a análise automatizada de dados pode contribuir para a eficiência clínica e a segurança do paciente, ao reduzir a carga cognitiva relacionada ao monitoramento de rotina. No entanto, os autores enfatizam que as recomendações geradas por IA devem ser submetidas à supervisão rigorosa de profissionais qualificados e ao julgamento clínico criterioso antes de serem incorporadas à prática assistencial padrão.

Diversas aplicações da IA na enfermagem já foram identificadas, com modelos de aprendizado de máquina sendo empregados, atualmente, na previsão de quedas, na avaliação do risco de lesões por pressão e na coordenação do cuidado. Tais tecnologias apresentam potencial significativo para otimizar fluxos de trabalho clínico e aprimorar os desfechos em saúde, tanto em contextos de cuidados agudos quanto na atenção comunitária. Entretanto, o número limitado de estudos prospectivos e a escassez de validações em cenários reais reforçam a necessidade urgente de investigações adicionais que assegurem a confiabilidade clínica e a segurança dessas ferramentas emergentes ^(2,5,6)^.

No campo do processamento de linguagem natural (PLN), Hossain et al. (2023)^(3)^ investigam a extração de informações clínicas a partir de prontuários eletrônicos de saúde (PES). Seus resultados demonstram que o PLN pode converter, de forma eficiente, textos clínicos não estruturados-como anotações de enfermagem e históricos de pacientes-em dados acionáveis. Ao automatizar a interpretação de registros em linguagem livre, os sistemas de IA contribuem para a redução de encargos administrativos e permitem que enfermeiras(os) concentrem sua atenção na tomada de decisão clínica. Ainda assim, o estudo salienta desafios persistentes, como o desequilíbrio dos dados e a necessidade de curadoria rigorosa, que devem ser enfrentados para garantir a fidedignidade e a precisão das saídas geradas por esses modelos ^(2,3,7)^.

É premente que exploremos as oportunidades e desafios associados à integração da IA na educação e na prática da enfermagem, com ênfase no potencial dos sistemas de aprendizagem personalizada para adaptar conteúdos às necessidades individuais de aprendizagem, promovendo, assim, melhor desempenho acadêmico e clínico. Embora a IA possa oferecer suporte à automatização de tarefas como a documentação e a alocação de recursos, reitera-se que a responsabilidade última pelo cuidado em saúde deve permanecer com profissionais devidamente habilitados. Tal perspectiva reforça a necessidade de formação continuada e da formulação de diretrizes éticas robustas, a fim de garantir a segurança do paciente e preservar a integridade da tomada de decisão clínica ^(1)^.

Porcellato, et al^(6)^ apresentam uma revisão sistemática das aplicações da IA na enfermagem em cuidados críticos, destacando o uso de técnicas de aprendizado profundo e aprendizado de máquina para prever complicações, apoiar processos de triagem e monitorar sinais vitais críticos em unidades de terapia intensiva (UTI) e serviços de emergência. Embora tais modelos demonstrem níveis promissores de acurácia, a heterogeneidade dos desenhos metodológicos e das fontes de dados limita a generalização dos achados. Os autores defendem a realização de estudos intervencionais robustos para avaliar, de forma acurada, o impacto real da IA sobre desfechos sensíveis à enfermagem^(3,6)^.

Topol^(2)^ oferece uma perspectiva visionária sobre a medicina de alto desempenho, ao enfatizar a convergência entre a inteligência humana e a artificial. O autor descreve como sistemas modernos de IA são capazes de processar imagens médicas de alta resolução, dados contínuos provenientes de biossensores e informações genômicas com velocidade e precisão excepcionais. Contudo, ressalta que a interpretação dessas informações continua a depender da expertise humana. Embora a IA possa aumentar a acurácia diagnóstica e a eficiência operacional, Topol destaca preocupações persistentes relacionadas ao viés algorítmico, à privacidade dos dados e à natureza opaca (“caixa preta”) dos sistemas de aprendizado profundo. Ele defende um modelo híbrido, em que o julgamento humano e os insights gerados por IA sejam integrados, explorando, assim, as potencialidades complementares de ambas as inteligências ^(2,3,5)^.

Passerini, et al^(8)^ ressaltam a importância de promover a colaboração eficaz entre seres humanos e modelos de linguagem de larga escala (LLMs). Embora esses modelos apresentem desempenho expressivo em diversas tarefas linguísticas, os autores apontam limitações significativas, como alucinações, inconsistências e respostas bajuladoras. Paralelamente, modelos multimodais emergentes (LMMs) começam a ampliar essas capacidades ao integrar análises visuais na tomada de decisão clínica-como, por exemplo, a interpretação da imagem de uma cicatriz para subsidiar um plano terapêutico. O estudo defende o desenvolvimento de estruturas de interação humano-IA, nas quais profissionais clínicos desempenhem papel central na orientação e validação das saídas geradas por IA, a fim de fortalecer a tomada de decisão clínica sem promover dependência excessiva da automação ^(2,7,8)^.

Ressaltamos que as ferramentas de IA têm o potencial de aprimorar significativamente a tomada de decisão clínica ao processar dados complexos e gerar insights que reforcem o julgamento reflexivo. Ainda que possam fortalecer decisões baseadas em dados, tais tecnologias devem operar em um arcabouço que preserve a autoridade, a autonomia e o pensamento crítico do(a) profissional de saúde. Validação rigorosa e monitoramento contínuo são essenciais para garantir a precisão, mitigar riscos e prevenir erros ^(9)^.

No contexto da enfermagem brasileira, a IA-especialmente os modelos de PLN, como o ChatGPT-pode contribuir para o processo de enfermagem e a melhoria da eficiência administrativa. Contudo, reiteramos que a responsabilidade clínica deve permanecer exclusivamente com profissionais legalmente habilitados. Alertamos para os riscos da delegação de responsabilidades clínicas a sistemas automatizados e defendemos a inclusão da educação em IA nos currículos de graduação e pós-graduação em enfermagem, a fim de desenvolver tanto a proficiência tecnológica quanto a capacidade de julgamento clínico ^(4)^.

Coletivamente, os estudos analisados evidenciam o potencial transformador da IA na enfermagem, em distintos contextos assistenciais. Em ambientes hospitalares, a IA pode automatizar a análise de dados, otimizar sistemas de triagem e apoiar decisões diagnósticas rápidas, promovendo eficiência operacional e permitindo que enfermeiras(os) concentrem-se em intervenções clínicas de maior complexidade. Na atenção primária, ferramentas baseadas em IA podem analisar PES e prever tendências de saúde, subsidiando o cuidado preventivo e a gestão individualizada de pacientes. Apesar dessas aplicações promissoras, uma lacuna crítica permanece: a maior parte das pesquisas ainda é retrospectiva ou experimental, com validação limitada em cenários clínicos reais. Ademais, os riscos inerentes a resultados imprecisos ou enviesados reforçam a necessidade de supervisão ativa e julgamento crítico por parte das(os) profissionais da saúde.

A integração da IA e dos LLMs na tomada de decisão em enfermagem representa uma fronteira promissora e em rápida expansão. Modelos avançados são capazes de fornecer avaliações preliminares de risco e resumos concisos de dados, possibilitando que profissionais de saúde dediquem maior atenção a decisões complexas e de alto impacto. Avanços recentes em modelos de raciocínio-como as séries ChatGPT o3, DeepSeek R1 e Gemini Flash Thinking-oferecem melhor compreensão contextual e capacidades sofisticadas de inferência, com potencial para transformar a interpretação e o uso de dados clínicos. No entanto, tais inovações ainda requerem validação prospectiva rigorosa antes de sua adoção segura na prática clínica. À medida que essas tecnologias continuam a evoluir, a colaboração humano-IA deve ser concebida de forma intencional, visando aproveitar o poder computacional da IA sem comprometer dimensões humanas essenciais, como a empatia, o julgamento ético e o cuidado individualizado. Assim, enfatizamos a importância de incorporar a alfabetização digital em IA nos programas de formação profissional e instamos os sistemas de saúde a desenvolverem diretrizes claras, baseadas em evidências, que assegurem o uso responsável e efetivo dessas ferramentas.

Reconhecemos que a integração da IA à prática clínica apresenta desafios éticos, legais e curatoriais que devem ser enfrentados com rigor. A mitigação de vieses algorítmicos, a proteção da privacidade dos dados e o esclarecimento das implicações legais são fundamentais para a implementação segura e responsável da IA na enfermagem. Garantir a explicabilidade e a transparência dos modelos de “caixa preta” é essencial para manter a confiança nos sistemas de IA e em seus resultados. Igualmente importante é a curadoria rigorosa dos dados utilizados para treinamento, a fim de minimizar vieses e salvaguardar a confidencialidade dos pacientes. Somente por meio da criação de marcos regulatórios consistentes e da validação contínua será possível assegurar que os sistemas de IA gerem resultados confiáveis, imparciais e interpretáveis-em apoio, e não em detrimento, da qualidade e da segurança da assistência ao paciente.

Em conclusão, a inteligência artificial oferece oportunidades substanciais para ampliar a eficiência, reduzir encargos administrativos e melhorar os desfechos clínicos, tanto em ambientes hospitalares quanto na atenção primária. Entretanto, sua implementação segura e eficaz depende de pesquisas prospectivas robustas, curadoria criteriosa de dados e supervisão profissional contínua. Superar a dicotomia entre eficiência automatizada e excelência clínica requer o enfrentamento de desafios éticos cruciais-como a mitigação de vieses, a proteção da privacidade de dados e a responsabilidade legal-, além de uma abordagem transparente quanto às limitações dos modelos de “caixa preta”. Adotar um modelo híbrido, em que a IA complemente, mas não substitua, o raciocínio clínico humano, é fundamental para garantir que essas tecnologias fortaleçam, e não comprometam, a qualidade e a integridade do cuidado em enfermagem.

# Translation: Inteligencia artificial en enfermería: avances en el juicio clínico y la toma de decisiones

**DOI:** 10.1590/0034-7167.2025780401es

**Authors:** Luciano Magalhães Vitorino, Gerson Hiroshi Yoshinari, Luís Carlos Lopes-Júnior

**Affiliations:** I Faculdade de Medicina de Itajubá. Itajubá, Minas Gerais, Brasil; II Universidade Federal do Espírito Santo. Vitória, Espírito Santo, Brasil

La rápida evolución de la inteligencia artificial (IA) ha transformado el campo de la atención médica al automatizar tareas rutinarias, optimizar los flujos de trabajo y mejorar la toma de decisiones clínicas. Los modelos computacionales avanzados demuestran la capacidad de analizar grandes conjuntos de datos clínicos y generar recomendaciones basadas en evidencia en tiempo real. Sin embargo, a pesar del desempeño prometedor en entornos de investigación controlados, todavía existe una brecha importante con respecto a la validación prospectiva de aplicaciones de IA en contextos de práctica de enfermería reales. La literatura actual sugiere que, si bien la IA tiene un potencial considerable para ayudar a las enfermeras a resolver desafíos tanto en entornos hospitalarios como de atención primaria de salud, las habilidades humanas esenciales, como el razonamiento clínico y la toma de decisiones reflexiva, siguen siendo irremplazables^(1-4)^.

Rony *et al*.^(5)^ demuestran que los algoritmos de IA pueden utilizarse para monitorizar los signos vitales y detectar signos tempranos de deterioro clínico, permitiendo así a los profesionales de enfermería dedicar más tiempo a la atención directa al paciente. Sus hallazgos muestran que el análisis de datos automatizado puede contribuir a la eficiencia clínica y la seguridad del paciente al reducir la carga cognitiva relacionada con el monitoreo de rutina. Sin embargo, los autores enfatizan que las recomendaciones generadas por IA deben estar sujetas a una supervisión rigurosa por parte de profesionales calificados y a un juicio clínico cuidadoso antes de incorporarse a la práctica de atención estándar.

Ya se han identificado varias aplicaciones de la IA en enfermería y actualmente se utilizan modelos de aprendizaje automático para predecir caídas, evaluar el riesgo de lesiones por presión y coordinar la atención. Estas tecnologías tienen un potencial significativo para optimizar los flujos de trabajo clínicos y mejorar los resultados de salud tanto en entornos de atención aguda como de atención comunitaria. Sin embargo, el número limitado de estudios prospectivos y la escasez de validaciones en entornos del mundo real refuerzan la necesidad urgente de investigaciones adicionales para garantizar la confiabilidad clínica y la seguridad de estas herramientas emergentes^(2,5,6)^.

En el campo del procesamiento del lenguaje natural (PLN), Hossain *et al*.^(3)^ investigan la extracción de información clínica de los registros médicos electrónicos (RME). Sus resultados demuestran que el PNL puede convertir eficientemente textos clínicos no estructurados, como notas de enfermería e historias clínicas de pacientes, en datos procesables. Al automatizar la interpretación de registros en lenguaje libre, los sistemas de IA ayudan a reducir las cargas administrativas y permiten a las enfermeras centrar su atención en la toma de decisiones clínicas. Aun así, el estudio destaca desafíos persistentes, como el desequilibrio de datos y la necesidad de una curación rigurosa, que deben abordarse para garantizar la confiabilidad y precisión de los resultados generados por estos modelos^(2,3,7)^.

Es urgente que exploremos las oportunidades y los desafíos asociados con la integración de la IA en la educación y la práctica de enfermería, con énfasis en el potencial de los sistemas de aprendizaje personalizados para adaptar el contenido a las necesidades de aprendizaje individuales, promoviendo así un mejor desempeño académico y clínico. Si bien la IA puede respaldar la automatización de tareas como la documentación y la asignación de recursos, se reitera que la responsabilidad final de la atención médica debe recaer en profesionales debidamente capacitados. Esta perspectiva refuerza la necesidad de formación continua y de formulación de directrices éticas sólidas para garantizar la seguridad del paciente y preservar la integridad de la toma de decisiones clínicas^(1)^.

Porcellato *et al*.^(6)^ presentan una revisión sistemática de las aplicaciones de IA en enfermería de cuidados críticos, destacando el uso de técnicas de aprendizaje profundo y aprendizaje automático para predecir complicaciones, apoyar procesos de triaje y monitorear signos vitales críticos en Unidades de Cuidados Intensivos y departamentos de emergencia. Aunque estos modelos demuestran niveles prometedores de precisión, la heterogeneidad de los diseños metodológicos y las fuentes de datos limita la generalización de los hallazgos. Los autores abogan por estudios de intervención sólidos para evaluar con precisión el impacto real de la IA en los resultados sensibles a la enfermería^(3,6)^.

Topol^(2)^ ofrece una perspectiva visionaria sobre la medicina de alto rendimiento al enfatizar la convergencia entre la inteligencia humana y artificial. El autor describe cómo los sistemas de IA modernos son capaces de procesar imágenes médicas de alta resolución, datos continuos de biosensores e información genómica con una velocidad y precisión excepcionales. Sin embargo, es importante señalar que la interpretación de esta información sigue dependiendo de la experiencia humana. Si bien la IA puede aumentar la precisión del diagnóstico y la eficiencia operativa, Topol destaca preocupaciones persistentes relacionadas con el sesgo algorítmico, la privacidad de los datos y la naturaleza opaca (“caja negra”) de los sistemas de aprendizaje profundo. Aboga por un modelo híbrido, en el que se integren el juicio humano y los conocimientos generados por la IA, explorando así las potencialidades complementarias de ambas inteligencias^(2,3,5)^.

Passerini *et al*.^(8)^ destacan la importancia de promover una colaboración eficaz entre los humanos y los modelos de lenguaje a gran escala (LLMs). Aunque estos modelos funcionan de manera impresionante en varias tareas lingüísticas, los autores señalan limitaciones significativas, como alucinaciones, inconsistencias y respuestas halagadoras. Paralelamente, los modelos multimodales emergentes (LMMs) están comenzando a ampliar estas capacidades al integrar análisis visuales en la toma de decisiones clínicas, como la interpretación de una imagen de una cicatriz para respaldar un plan terapéutico. El estudio aboga por el desarrollo de marcos de interacción humano-IA en los que los profesionales clínicos desempeñen un papel central a la hora de guiar y validar los resultados generados por la IA, con el fin de fortalecer la toma de decisiones clínicas sin promover una dependencia excesiva de la automatización^(2,7,8)^.

Destacamos que las herramientas de IA tienen el potencial de mejorar significativamente la toma de decisiones clínicas al procesar datos complejos y generar conocimientos que refuerzan el juicio reflexivo. Si bien pueden posibilitar decisiones basadas en datos, dichas tecnologías deben operar dentro de un marco que preserve la autoridad, la autonomía y el pensamiento crítico de los profesionales de la salud. Una validación rigurosa y un seguimiento continuo son esenciales para garantizar la precisión, mitigar el riesgo y prevenir errores^(9)^.

En el contexto de la enfermería brasileña, la IA, especialmente los modelos de PNL como *ChatGPT*, pueden contribuir al proceso de enfermería y mejorar la eficiencia administrativa. Sin embargo, reiteramos que la responsabilidad clínica debe recaer exclusivamente en profesionales legalmente habilitados. Advertimos sobre los riesgos de delegar responsabilidades clínicas a sistemas automatizados y abogamos por la inclusión de la educación en IA en los planes de estudio de enfermería de pregrado y posgrado para desarrollar tanto la competencia tecnológica como las habilidades de juicio clínico^(4)^.

En conjunto, los estudios analizados demuestran el potencial transformador de la IA en enfermería en diferentes contextos de atención. En entornos hospitalarios, la IA puede automatizar el análisis de datos, optimizar los sistemas de triaje y respaldar decisiones de diagnóstico rápidas, promoviendo la eficiencia operativa y permitiendo que las enfermeras se concentren en intervenciones clínicas más complejas. En atención primaria, las herramientas basadas en IA pueden analizar los RME y predecir las tendencias de salud, lo que respalda la atención preventiva y la gestión individualizada de los pacientes. A pesar de estas aplicaciones prometedoras, aún existe una brecha crítica: la mayor parte de la investigación aún es retrospectiva o experimental, con una validación limitada en entornos clínicos reales. Además, los riesgos inherentes a los resultados inexactos o sesgados refuerzan la necesidad de una supervisión activa y un juicio crítico por parte de los profesionales de la salud.

La integración de la IA y los LLM en la toma de decisiones de enfermería representa una frontera prometedora y en rápida expansión. Los modelos avanzados son capaces de proporcionar evaluaciones preliminares de riesgos y resúmenes de datos concisos, lo que permite a los profesionales de la salud centrar más su atención en decisiones complejas y de alto impacto. Los avances recientes en modelos de razonamiento, como la serie *ChatGPT* o3, *DeepSeek* R1 y *Gemini Flash Thinking*, ofrecen una mejor comprensión contextual y capacidades de inferencia sofisticadas, con el potencial de transformar la interpretación y el uso de datos clínicos. Sin embargo, estas innovaciones aún requieren una rigurosa validación prospectiva antes de su adopción segura en la práctica clínica. A medida que estas tecnologías continúan evolucionando, la colaboración entre humanos e IA debe diseñarse intencionalmente para aprovechar el poder computacional de la IA sin comprometer dimensiones humanas esenciales como la empatía, el juicio ético y la atención individualizada. Por lo tanto, destacamos la importancia de incorporar la alfabetización digital en IA en los programas de capacitación profesional e instamos a los sistemas de salud a desarrollar pautas claras basadas en evidencia que aseguren el uso responsable y efectivo de estas herramientas.

Reconocemos que la integración de la IA en la práctica clínica presenta desafíos éticos, legales y curatoriales que deben abordarse rigurosamente. Mitigar los sesgos algorítmicos, proteger la privacidad de los datos y aclarar las implicaciones legales son fundamentales para la implementación segura y responsable de la IA en enfermería. Garantizar la explicabilidad y la transparencia de los modelos de “caja negra” es esencial para mantener la confianza en los sistemas de IA y sus resultados. Igualmente importante es la curación rigurosa de los datos utilizados para la capacitación con el fin de minimizar el sesgo y salvaguardar la confidencialidad del paciente. Solo mediante la creación de marcos regulatorios consistentes y una validación continua será posible garantizar que los sistemas de IA generen resultados confiables, imparciales e interpretables que respalden, y no perjudiquen, la calidad y la seguridad de la atención al paciente.

En conclusión, la IA ofrece oportunidades sustanciales para aumentar la eficiencia, reducir las cargas administrativas y mejorar los resultados clínicos tanto en entornos hospitalarios como de atención primaria. Sin embargo, su implementación segura y efectiva depende de una investigación prospectiva sólida, una conservación cuidadosa de los datos y una supervisión profesional constante. Superar la dicotomía entre la eficiencia automatizada y la excelencia clínica requiere abordar desafíos éticos cruciales, como la mitigación del sesgo, la protección de la privacidad de los datos y la responsabilidad legal, así como un enfoque transparente de las limitaciones de los modelos de “caja negra”. Adoptar un modelo híbrido, en el que la IA complemente, pero no reemplace, el razonamiento clínico humano, es fundamental para garantizar que estas tecnologías fortalezcan, en lugar de comprometer, la calidad y la integridad de la atención de enfermería.
